# Experimental Investigation on Mechanical and Thermal Properties of Concrete Using Waste Materials as an Aggregate Substitution

**DOI:** 10.3390/ma15051728

**Published:** 2022-02-25

**Authors:** Gavril Sosoi, Cherifa Abid, Marinela Barbuta, Andrei Burlacu, Marius Costel Balan, Marius Branoaea, Robert Stefan Vizitiu, Fabrice Rigollet

**Affiliations:** 1 Faculty of Civil Engineering and Building Services, “Gheorghe Asachi” Technical University of Iasi, 700050 Iasi, Romania; gavrilsosoi@gmail.com (G.S.); marinela.barbuta@academic.tuiasi.ro (M.B.); marius.branoaea@tuiasi.ro (M.B.); robert.vizitiu@tuiasi.ro (R.S.V.); 2 IUSTI UMR 7343, Aix-Marseille Université, 13453 Marseille, France; cherifa.abid@univ-amu.fr (C.A.); fabrice.rigollet@univ-amu.fr (F.R.)

**Keywords:** cement based concrete, ecological concrete, waste aggregates, compressive strength, thermal conductivity

## Abstract

The continuous growth of the concrete industry requires an increased quantity of cement and natural aggregates year after year, and it is responsible for a major part of the global CO_2_ emissions. These aspects led to rigorous research for suitable raw materials. Taking into account that these raw materials must have a sustainable character and also a low impact on environmental pollution, the replacement of the conventional components of concrete by residual waste can lead to these targets. This paper’s aim is to analyze the density, compressive strength and the thermal conductivity of nine concrete compositions with various rates of waste: four mixes with 10%, 20%, 40% and 60% chopped PET bottles aggregates and 10% fly ash as cement partial substitution; a mix with 60% waste polystyrene of 4–8 mm and 10% fly ash; a mix with 20% waste polystyrene of 4–8 mm, 10% waste polystyrene of 0–4 mm and 10% fly ash; a mix with 50% waste polystyrene of 4–8 mm, 20% waste polystyrene of 0–4 mm and 20% fly ash two mixes with 10% fly ash and 10% and 40% waste sawdust, respectively. Using 60% PET aggregates, 60% polystyrene granules of 4–8 mm, or 20% polystyrene of 0–4 mm together with 50% polystyrene of 4–8 mm led to the obtainment of lightweight concrete, with a density lower than 2000 kg/m^3^. These mixes also registered the best results from a thermal conductivity point of view, after the concrete mix with 40% saw dust. Regarding compressive strength, the mix with 10% PET obtained a result very close to the reference mix, while those with 20% PET, 40% PET, 30% polystyrene, and 10% saw dust, respectively, registered values between 22 MPa and 25 MPa, values appropriate for structural uses.

## 1. Introduction

Energy consumption in the world is increasing and, therefore, a need for taking into consideration the ongoing energy crisis and the impact it has on the environment is present. In terms of energy demand, the built environment is a net consumer of energy, demanding over 36% of the global energy, and upwards to 50% of raw materials worldwide. In terms of the environmental impact, buildings are responsible for over 39% of the global greenhouse gas emissions [1].

It is estimated that: 1 ton of Portland cement produces 0.96 tons of CO_2_ and 1 ton of concrete produces 0.108 tons CO_2_. According to experts, the concrete industry is responsible for 7 to 9% of the global greenhouse gas emissions [2,3].

The use of wastes as an aggregate substitution in construction materials represents a great alternative, especially in cement based concretes, due to large scale use in the construction industry. By using waste materials, such as chopped PET bottles, granular polystyrene, and waste wood, as aggregates in the concrete mixture, an ecological and performant concrete can be obtained, such as lightweight concrete, that can be used to reduce the weight of buildings. Another significant advantage of this type of concrete mixture is the valorification of waste materials that can produce a negative impact on the environment.

The purpose of this paper is to investigate the density, compressive strength and thermal conductivity of various concrete mixes using waste as aggregates and cement substitutions. The study implied the development of ten concrete mixes, which included a reference concrete mix made with conventional components only (river aggregates and cement), and nine concrete compositions with waste materials as aggregates and cement partial replacements, as follows: four mixes with 10%, 20%, 40% and 60% chopped PET bottles aggregates and 10% fly ash as cement partial substitution; a mix with 60% waste polystyrene of 4–8 mm and 10% fly ash; a mix with 20% waste polystyrene of 4–8 mm, 10% waste polystyrene of 0–4 mm and 10% fly ash; a mix with 50% waste polystyrene of 4–8 mm, 20% waste polystyrene of 0–4 mm and 20% fly ash two mixes with 10% fly ash and 10% and 40% waste sawdust, respectively. Using the waste materials mentioned can contribute significantly to reducing waste quantity and CO_2_ emissions, by decreasing the produced quantity of cement, and by natural aggregates’ use in a concrete mix.

If waste, such as chopped PET, granular polystyrene, and waste sawdust, are to be used as aggregates in the composition of concrete, then the pollution and impact of the construction sector on the environment will decrease, these facts represent a great advantage both in terms of recycling and reducing the consumption of natural ingredients, such as sand.

## 2. State of the Art

There are various recent studies on the use of waste chopped polyethylene terephthalate, granular polystyrene, and waste wood (sawdust) in the composition of the concrete mixture by replacing natural aggregates [4,5,6,7,8,9,10,11].

Furthermore, by investigating the mechanical properties of the concrete with waste chopped PET bottles, granular polystyrene, and waste wood as a substitution in the concrete mix, researchers obtained results that highlight the fact that the mechanical properties of fresh and hardened concrete are being influenced by the ratio of the replaced material in the concrete mix [12,13,14,15,16,17,18,19,20,21].

In terms of research trends, scientist have analyzed the incorporation of various materials into concrete in order to reduce the required raw materials, ranging from crushed recovered concrete and ceramics, vegetal byproducts and even plastics. Some examples of such studies are presented in Table 1.

### 2.1. Concrete with PET and Polystyrene

The use of PET and polystyrene in concrete mixtures, both in structural or lightweight concrete, was analyzed in a number of studies with the aim to reduce the waste in the world and reduce the quantity of required raw materials. The analyzed properties of these concretes include—but are not limited to—compressive strength, density, tensile strength, elasticity modulus, flexural strength, durability, thermal properties and fire resistance.

In their study on concrete with polystyrene with a ratio ranging from 20% to 80% of the total volume and a resin mixture of 0.5%, 1%, and 1.5% of the total weight, Kaya and Kar concluded that, as the added components percentage increase, the porosity of the concrete increases and the mechanical properties, such as density, tensile strength, compressive strength, and thermal conductivity, decrease [11].

In their research, Saikia and Brito performed an experimental investigation on specimens for compressive strength, split tensile strength, and flexural strength, observing that an increase in the concentration of the PET ratio as aggregate in the mixture leads to a decrease in the compressive strength, split tensile strength and flexural strength of the concrete [12].

The study published by Rahmani et al. investigated the mechanical properties of cubic and cylindrical concrete specimens with 5% to 15% substitution of sand with particles of PET and different water to cement ratios. The experimental study revealed that the compressive and flexural strength initially tend to increase, however, they tend to decrease after a period of time. Moreover, the experiments highlighted that the samples containing PET particles have a lower density, splitting tensile strength, modulus of elasticity, and workability [13].

Jahidul et al. carried out an experimental investigation on the properties of PET aggregate concrete by replacing between 0 and 50% of the volume of coarse aggregates, observing that this type of concrete provided better workability than regular concrete while using a similar w/c ratio, the density of the PET concrete was reduced by 4 to 10%, thus reducing the self-weight of the structural element. The optimal substitution ratio was 20%, this percentage ensuring comparable compressive strength with natural aggregate concrete [15].

Maldonado-Bandala et al. observed similar results regarding mechanical parameters such as density and compressive strength—that these parameters decrease as the ratio of polystyrene increases—but the experiment revealed that the corrosion protection of the concrete with polystyrene is greatly superior to conventional concrete [16].

Studies performed by Sayadi et al. revealed that adding expanded polystyrene into the concrete mixture translates to a decrease in thermal parameters such as thermal conductivity and fire resistance [17].

### 2.2. Concrete with Sawdust

Sawdust, as a byproduct of the wood industry, has been researched and implemented in various forms since the 20th century in regions such as the United State of America, Asia (Malaysia and Singapore) and Europe (Germany and the UK). The possible applications of this material are significant, as highlighted by the fact that this material, in various forms and adaptations, has been used for building elements such as walls and floors [32].

Sales et al. analyzed the incorporation of untreated wood shavings into concrete to obtain lightweight concrete. The study highlighted that, through the introduction of sawdust into the concrete mixture, some key parameters were improved: the thermal conductivity of the concrete was improved and the mass density was reduced. At the same time, the sawdust presented a good adherence to the concrete matrix and the structure of the mixture was homogeneous [33].

Sofi et al. investigated the compressive strength of concrete samples with a volume of added sawdust ranging from 0% to 15% in the concrete mix at two curing periods, at 7 and 28 days. The results obtained after the curing periods show that the compressive strength is higher for concrete samples with a lower substitution percentage of sawdust [18].

The implementation of sawdust in concrete has not reached the level of widespread adoption due to the limitations of sawdust, which has low compressive strength. At the same time, the advantages of concrete with sawdust (reduction in structural weight and, as a consequence, reduced loads on the foundation, reduced damage phenomena and increased the lifespan of the structure, along with easier handling and reduced consumption of raw materials) justify the increasing interest in this type of material.

### 2.3. Concrete with Fly Ash

Fly ash is a byproduct of burning coal in power plants in the form of ultrafine solid residue. In numerous developed and developing countries, such as US, China and India, where coal is used, it is one of the largest solid wastes. Fly ash has been implemented as an additive in concrete since the mid-20th century as a replacement for some raw materials such as cement or as a mixture with a clinker [34].

The analysis performed by multiple researchers highlights the increase in interest towards ecological concrete and its potential to reduce the carbon footprint buildings impose on the environment. Even though some mechanical parameters are not as high with the substitution of natural aggregates, these types of walls can be applied in nonstructural elements.

## 3. Experimental Works

### 3.1. Materials

#### 3.1.1. Cement and Fly Ash

The cement used in the concrete mixture is Portland cement type CEM II 45.2 R produced in Romania following the EN 197-1 standard, this fact is evidenced both from the producer’s multiple declarations of conformity, performance certificates and through independent tests conducted on a cement sample. The cement has the following components: Portland clinker maximum dosage 94%, limestone 6–20%, and auxiliary components 0–5% [35,36].

**Figure 1:**
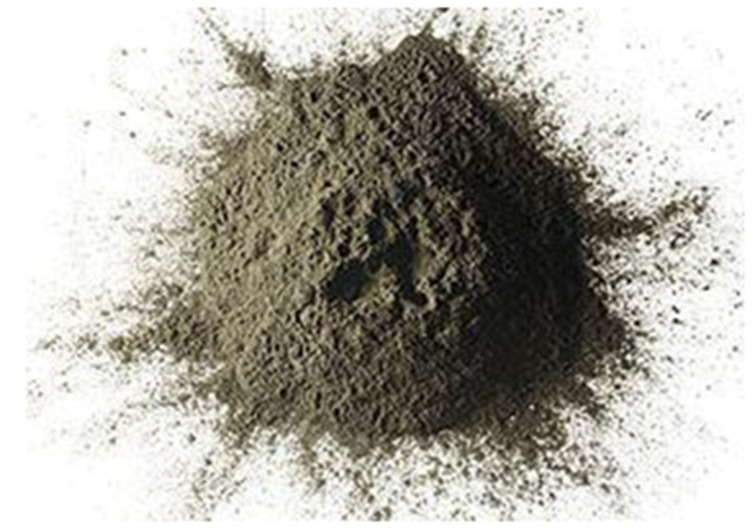
Fly ash from Holboca Electric Power Plant.

The fly ash used as a replacement for the cement, which is released with the exhaust fuel gases, presented in Figure 1 is a byproduct from the Holboca Electric Power Plant in Iasi, with a bulk density ranging from 2400 to 2550 kg/m^3^, specific surface area of 520 m^2^/kg, particle size ranging from 0.01 to 400 µm and, as main chemical components, O_2_ (43.3%), Si (30.8%), Al (19.2%) and Fe (3.05%) [37,38].

#### 3.1.2. Aggregates

**Figure 2:**
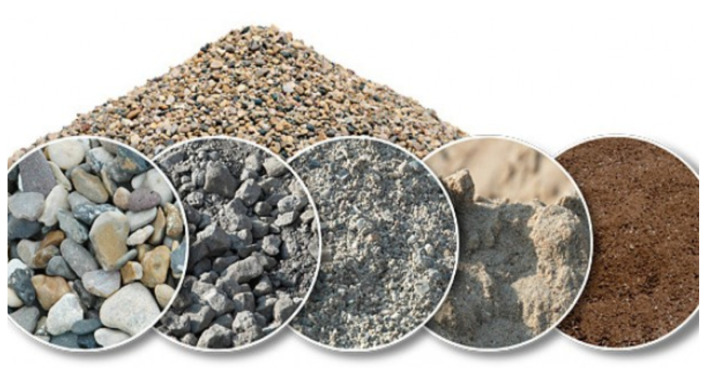
Natural aggregates used in concrete mix.

The aggregates used in the composition of the concrete can be classified into:

Natural aggregates such as sand with a specific gravity of 2.68, bulk density 1700 kg/m^3^, and absorption of 1%, and two sorts of river stone (sort I 4–8 mm and sort II 8-16 mm) with a specific gravity of 2.62, bulk density 1400 kg/m^3^, and absorption of 0.9%, presented in Figure 2.

**Figure 3:**
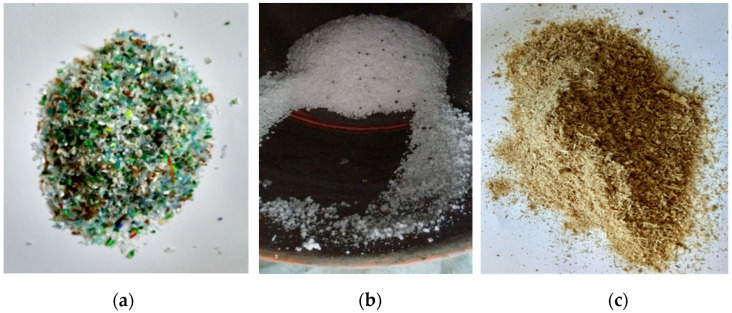
Waste aggregates used in the concrete mixture: (**a**) chopped PET bottle; (**b**) granular polystyrene; (**c**) sawdust/waste wood.

Waste aggregates, evidenced by Figure 3, such as:

- Chopped PET from bottles (having dimensions between 0–4 mm), resulting from cutting discarded polyethylene terephthalate (PET) bottles. After the cutting of the material it was sieved in order to obtain the desired particle dimensions. This waste material presents high stability and nonreactivity with substances. The unit weight of the chopped PET was determined experimentally and has a value of 433 kg/m^3^ [39];
- Granular polystyrene (with dimensions 0–4 mm and 4–8 mm), which can be reused in the shape of granules, resulting from shredding, cutting or thermal treatment;
- Waste wood/sawdust (with dimensions 0–4 mm), is a byproduct resulting from the wood industry. The collected sawdust was air dried and afterwards sieved, in order to imitate the size of the natural aggregates. Sawdust in terms of chemical composition is comprised of carbon (60.8%), hydrogen (5.2%), oxygen (33.8%), and nitrogen (0.9%). The primary components of dry wood are cellulose, lignin, hemicelluloses, and small amounts (5–10%) of extraneous materials. The unit weight of the sawdust was determined experimentally and has a value of 168 kg/m^3^ [39,40].

### 3.2. Concrete Mixture

The concrete mixture without any substitutions was made according to the following recipe: sand 803 kg/m^3^, river gravel with 4–8 mm in diameter 384 kg/m^3^, river gravel with 8-16 mm in diameter 559 kg/m^3^, cement 360 kg/m^3^, water 180 l/m^3^ and a 1% superplasticizer additive of the cement mass.

In order to conduct the experiments and determine the mechanical parameters and the advantages and disadvantages of the partial substitution of the natural aggregate with either chopped PET, granular polystyrene, or waste wood (sawdust), nine types of different concrete mixtures were considered, with the mixing proportions and the quantities of the used materials presented in Table 2. The concrete was casted into 10 cubic shape specimens and 6 plate shape specimens for each concrete mixture.

The concrete mixture realization process involved adding the materials and water in the two-blade mixer, the mixing process was finished after 30–40 min.

Samples S1 to S4 also contain fly ash, which replaced 10% of the cement, and from 10% to 60% chopped PET replacing the same mass volume of cement.

In the concrete mix using waste granular polystyrene, three samples in which 10% of cement was replaced with fly ash, and the substitution of natural aggregates used were replaced by 60% of the sort I aggregate mass 4–8 mm were replaced with granular polystyrene 4–8 mm, resulting the concrete sample notated S5.

Following this, the concrete mixture was changed once again by replacing two natural aggregates in order to create sample S6, as follows: 90% of the sand was used, the remaining 10% being substituted by 10% of the sand mass with granular polystyrene 0-4 mm in volume, and, as a secondary substitution, only 80% of the original sort I, with a size of the granules of 4–8 mm, 20% from the sort I mass was replaced with granular polystyrene 4–8 mm in volume and a further substitution of 10% of the cement was made with fly ash.

For the concrete sample S7, another modification in the concrete mixture was made, in which 20% of the cement was replaced with fly ash and two more natural aggregates were partially replaced with granular polystyrene, replacing 20% of the sand mass with granular polystyrene 0–4 mm in volume and 50% of the sort I mass 4–8 mm was replaced by 50% granular polystyrene 4–8 mm in volume.

The last two samples, S8 and S9, were realized by further modifying the mixture: 10% of the cement was replaced with fly ash in both concrete samples and a percentage of the sand was replaced with its corresponding volume of sawdust. In the case of sample S8 only 10% of the sand was replaced and in the case of sample S9, 40% of the sand mass was replaced with the same volume of sawdust.

### 3.3. Preparation of Concrete Samples

**Figure 4:**
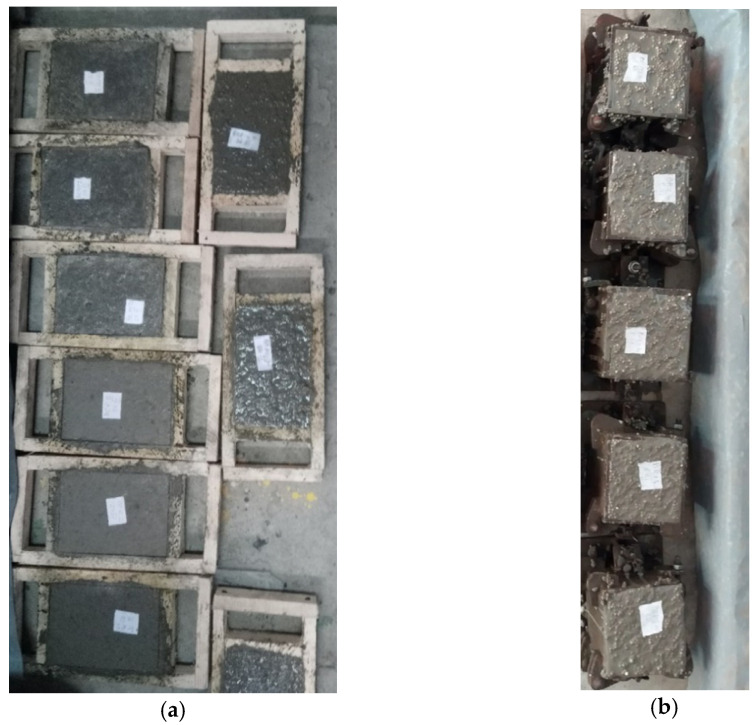
View of concrete samples: (**a**) cubic shape concrete samples, (**b**) plate shape concrete samples.

Following the mixing process, the concrete mixtures were poured into cubic shapes with the side length of 150 mm, Figure 4a, and rectangular prismatic shapes with the following dimensions 150 mm in length, 120 m width, and 20 mm depth, Figure 4b, in order to perform the experimental measurements.

The concrete samples in cubic shapes were made for the experiment of the compressive strength and density investigation; the rectangular prismatic concrete samples (plate concrete samples) were made for the experiment of the heat transfer coefficient investigation.

After the preparation, the concrete samples were left for curing for 28 days in laboratory conditions at a controlled temperature.

## 4. Characterization of Concrete Properties

### 4.1. Density

Experimental investigation on the density of the concrete samples was carried out according to the EN12390-7 standard [35] on tree specimens for every type of concrete mix with waste substitution by determining the mass and the volume of the specimens. The volume was determined with the reference method by water displacement after ensuring that the specimens were in saturated conditions. The mass in water was measured by raising the water level from a tank until the stirrup without the specimens was touching the bottom of the tank. After this procedure, the water surface surplus was removed by wiping the specimens and the mass of the specimens in air conditions was measured. With the values obtained we calculated the volume of the specimens with the Formula (1):

(1) $$V=\frac{m_{a}-\left[\left(m_{st}+m_{w}\right)-m_{st}\right]}{\rho_{w}}$$

The mass of the specimens was measured after the curing period, according to the EN12390-7 standard 35.

The density can be calculated with the formula:

(2) $$\rho =\frac{m}{V}$$

### 4.2. Compressive Strength

The preparing and keeping of the concrete samples were performed respecting the indications of EN 12390-2 standard [41]. Following the standard instructions the concrete samples were cured for a period of 28 days. During the curing period the samples were kept in water for 7 days after that in dry conditions at constant temperature of 20 °C with the maximum tolerance ±2 °C.

The compressive strength was determined according to EN 12390-3 standard [42] on tree specimens for every type of concrete mix with waste substitution on the hardened cubic concrete samples with the edge of 150 mm. The samples were tested after the curing period with a press having a maximum load force of 2000 kN. The preparation of the specimen for the experimental investigation on compressive strength was realized by centering the specimen on the lower platen.

**Figure 5:**
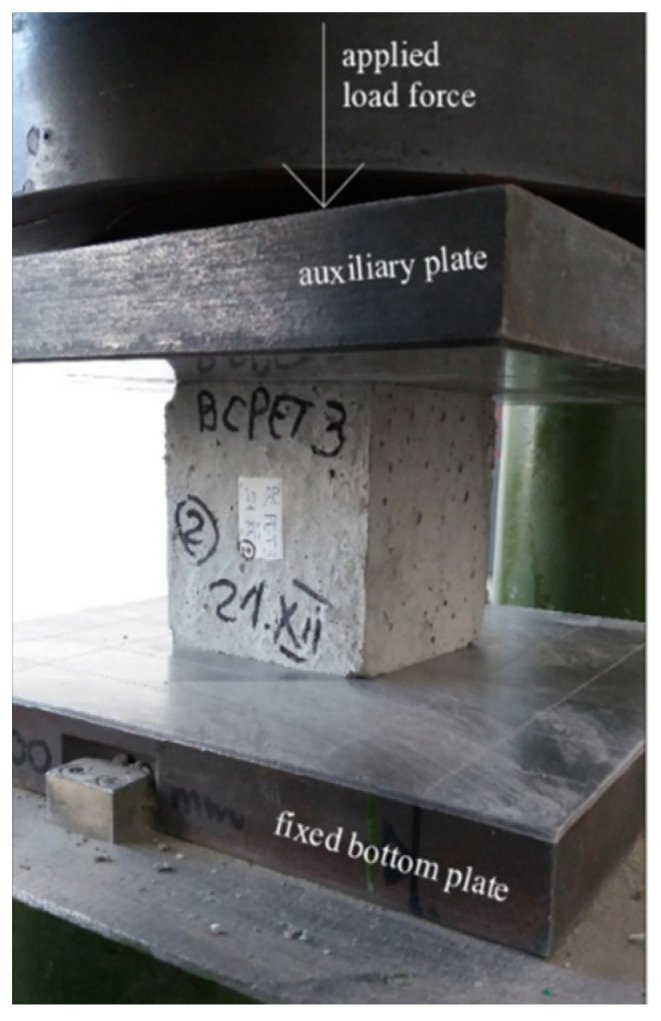
Experimental test of the compressive strength.

In the testing procedure presented in Figure 5, a first initial rate load force of 0.6 N/mm^2^·s constant at the beginning is applied on the area of the concrete sample without shock, after applying the first load force to the specimen, the load was increased continuously at a constant rate ±10% until the specimen reached the maximum load failure.

### 4.3. Discussion and Analysis

**Figure 6:**
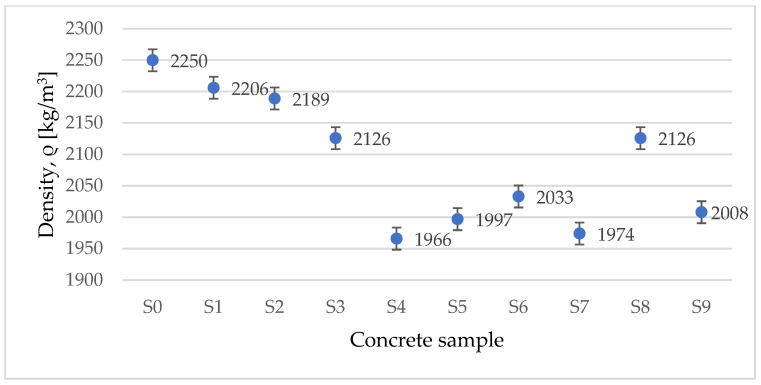
Variation of density for tested concrete samples.

The experimental determination of the concrete density is presented in Figure 6. From the measurements, the fact that an increase in the concentration of added waste aggregates leads to a decrease in the concrete density. For the obtained values for the densities of the concrete samples with waste as aggregate substitution, the uncertainty on the results calculated for the density is from 3.6% to 36.88%.

In the testing procedure presented in Figure 6, an initial load force of 0.6 N/mm^2^·s constant force is applied at the beginning on the surface area of the concrete sample without shock. After applying the first load force to the specimen, the load was increased continuously at a constant rate of ±10% until the specimen reached the maximum load failure. The uncertainty of the obtained results was calculated for the compressive strength on the studied specimens and it resulted in a minimum of 0.3% and a maximum value of 1.35%.

**Figure 7:**
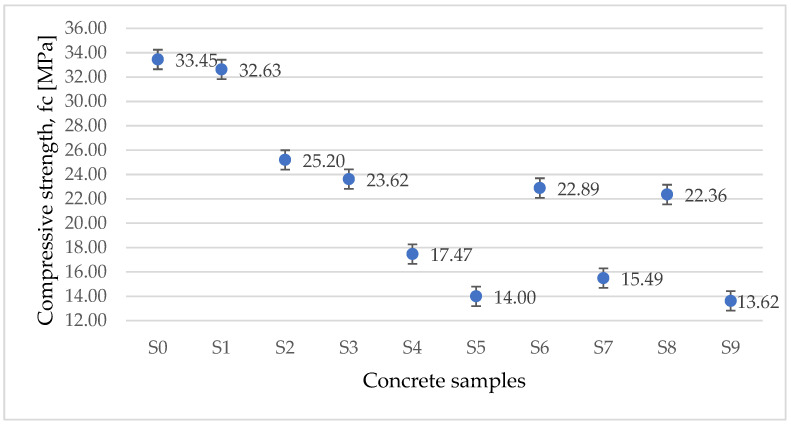
Variation of compressive strength for tested concrete samples.

The variation of compressive strength presented in Figure 7 represents the average of the measured results on tree concrete specimens. As we expected, the compressive strength is decreasing by using even a small percentage of waste as a substitution.

**Figure 8:**
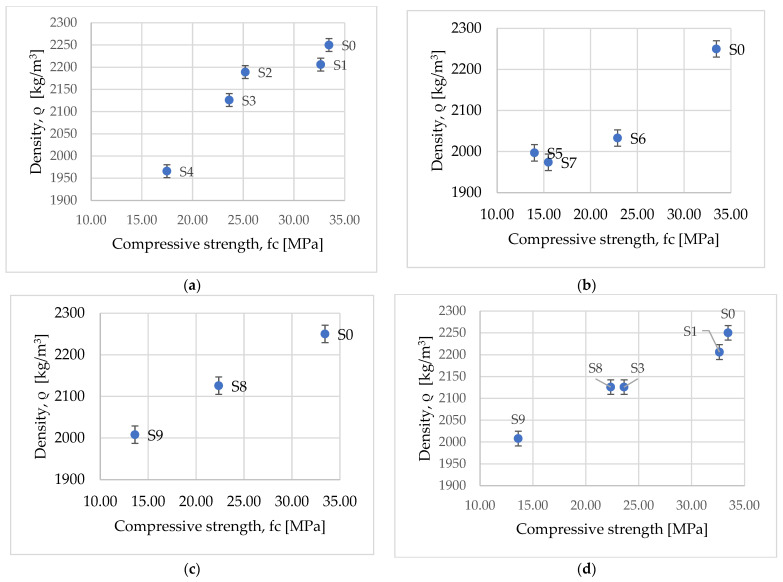
Variation of density versus compressive strength for concrete with waste: (**a**) concrete with waste chopped PET; (**b**) concrete with granular polystyrene; (**c**) concrete with sawdust; (**d**) concrete with waste chopped PET and sawdust.

In Figure 8a–d the variation of the density versus compressive strength of the concrete samples with waste substitution compared with the concrete sample with no waste replacement was analyzed.

A first analysis was performed by comparing the density versus compressive strength of concretes in which one natural aggregate was replaced with the same waste used in the concrete mixture, in this case, the natural aggregate sand was substituted with waste chopped PET.

By comparing the obtained results Figure 8a, in which only one natural aggregate was replaced, we observed that, up to a ratio of 20% substitution of natural aggregate with waste chopped PET, the results of the compressive strength show that the concrete can be used as a structural element and, due to it having a relatively high density, the concrete can be used as a heat-accumulating wall. Meanwhile, by increasing the waste substitution up to 60%, we observed that the compressive strength and density decrease, with the result that the concrete structure can be used as a self-sustained material or even as a heat-accumulating wall.

The second analysis was carried out on the concrete samples, substituting the natural aggregates with granular polystyrene. The results presented in Figure 8b highlight the fact that, in the case where a high percentage of aggregate sand, the compressive strength is greatly reduced and the density is slightly lower in comparison to the results of concrete samples with two natural aggregates as a substitution. The types of concrete with a high percentage of aggregates substitution can be used as a self-sustained structure and thermal insulating material, while replacing a small percentage of the natural aggregates with waste. Continuing the analyses of the mechanical properties for the concrete samples with waste wood substitution, Figure 8c, the results show that a higher percentage of substitution has the same effect as in the case of using waste chopped PET and granular polystyrene.

A final analysis on the mechanical properties for the concrete with different waste substitution at the same percentage as the waste replacement is presented in Figure 8d, which highlights that the concrete has the same density at 40% substitution with waste chopped PET as at 10% of the substitution with sawdust, resulting that the waste wood is negatively affecting the mechanical properties even at a reduced percentage of substitution, the concrete could be used as self-sustained material or as heat accumulator wall.

Results from Figure 8 were obtained after testing three specimens for the same concrete mix with waste aggregates. The values presented in Figure 8 were obtained by calculating the average of the measured values obtained at the end of the test.

## 5. Characterization of Concrete Thermal Properties

### 5.1. Experimental Set Up and Process

**Figure 9:**
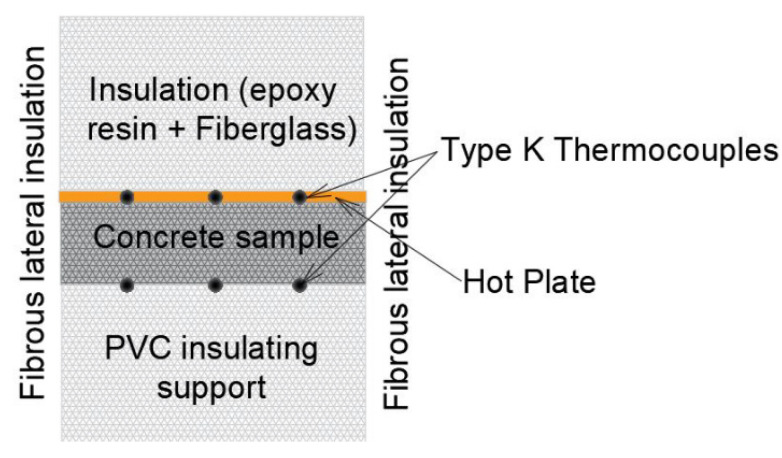
Experimental device.

The thermal properties were determined using an experimental device, developed at IUSTI Laboratory, which is based on the hot plane method with temperature measurements on both sides of the sample. The characterization is carried out at room temperature. A scheme of the experimental device is shown in Figure 9.

The concrete sample is heated in the front face by a heating sole (hot plane) (Figure 9) consisting of a flat electrical resistance pasted to a copper plate. This part of the sample is grooved in order to allow the placement of three K thermocouples in contact with the sample. Three other thermocouples are placed in grooves of the sample holder that will be in contact with the ‘rear face’ of the sample (Figure 9). A thermal paste provides good contact between the faces of the sample, the hot plate, and the sample holder. Thanks to the uniformity of the supplied heat flux and the limitation of the lateral thermal losses, the heat transfer in the sample are assumed to be 1D. For each test, the experimental parameters to adjust are the heat flux supplied to the hot plane, the heating duration, and the test duration. Thus, for these various samples, the duration of the heating is 6 min for a heat flux of 16 W.

To achieve these measurements, the concrete sample was first positioned in the thermal device (Figure 9), and then heated for 6 min; then the heating was stopped and the cooling of the sample was awaited until it reached its steady state. The temperature signals were recorded; the signals corresponding to the 3 K thermocouples in the front face of the sample (Figure 9) were averaged to provide the thermogram of the front face; the same process was performed for the signals given by the 3 K thermocouples at the rear face of the sample (Figure 9).

A transient thermal model (thermal quadrupoles) was used to calculate the theoretical thermogram of the rear face, by convolution of the thermogram of the front face of the sample. This theoretical thermogram is a function of the following parameters:

- t_d_ = e^2^/a
- a = k/ρCp
- x = b/b_PVC_, corresponding to the ratio of the samples upon the sample’s holder (PVC) effusivities.

The effusivity is given by:

- $b=\sqrt{k\rho c_{p}}=k/\sqrt{a}=\rho c_{p}\sqrt{a}$

These parameters were then identified by a least squares method, minimizing the gap between the theoretical and experimental thermograms, over range duration around the diffusive time.

The appropriate following parameters are then extracted.

- The thermal diffusivity a;
- The thermal effusivity b;
- The thermal conductivity $k=b\sqrt{a}$;
- The thermal capacity $c_{p}=b/\rho \sqrt{a}$.

The uncertainties calculation were carried out taking into account:

- The error of the thermocouples measurements (standard deviation: 0.005°C);
- The error due to the considered known parameters for determining a, b, k, ρ, and C_p_, i.e.,: error on the thickness e: 1%; error on the PVC effusivity b_PVC_: 10% and density error ρ: 4%.

First, a calibration was achieved to determine the sample holder (PVC) thermal diffusivity and effusivity. It was found that b_PVC_ is around (518 ± 10%) Jm^−2^K^−1^s^−0.5^, which corresponds to values given by the manufacturer.

### 5.2. Results and Discussion

**Figure 10:**
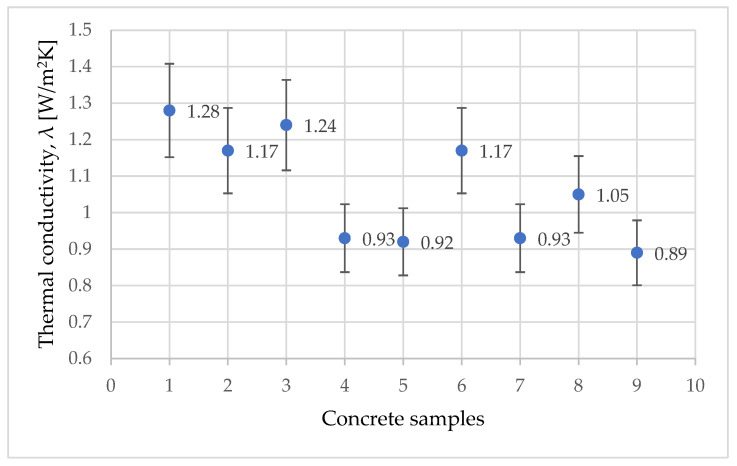
Variation of thermal conductivity.

For each sample, the same process was applied in order to determine the various thermal parameters. In Figure 10, the thermal conductivity is plotted for the various considered concrete samples.

From the thermal conductivity measurements, one can observe that the values of thermal conductivity are lower than the thermal conductivity of the standard concrete (S0). This result is consistent with the results found in the literature, since the thermal conductivity of the added waste is below the concrete one. Furthermore, reducing the thermal conductivity of concrete would provide better insulation for the building.

**Figure 11:**
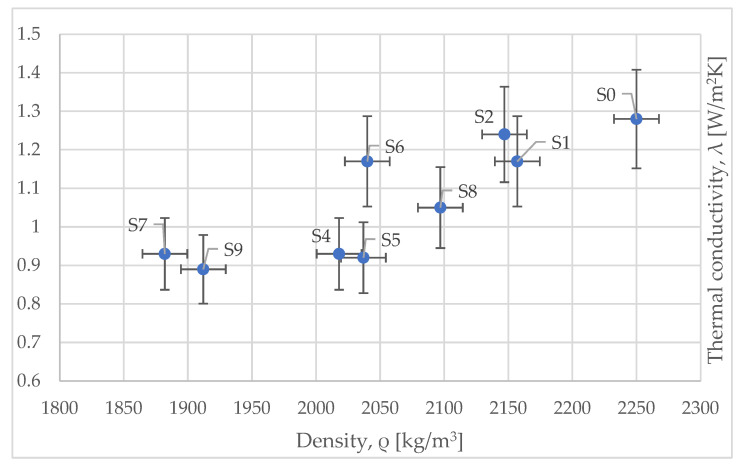
Variation of thermal conductivity versus density.

Following the measurements, Figure 11 was created highlighting the variation of thermal conductivity of the samples versus their density. As for compressive strength, the same behavior is highlighted; indeed, low density leads to low thermal conductivity.

**Figure 12:**
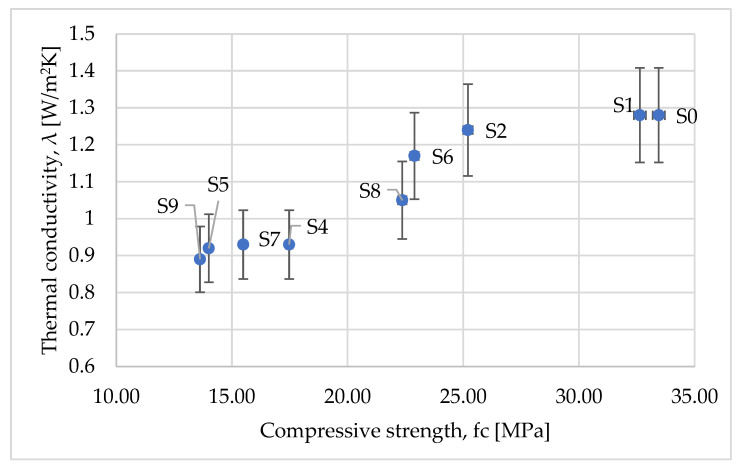
Variation of compressive strength versus density.

In order to illustrate the effect of the addition of waste on the global behavior of the concrete, another figure was created, Figure 12, which shows the compressive strength ratio versus the thermal conductivity.

From Figure 12, one can notice the existence of three zones:

- The first one corresponds to concrete formed by the addition of a high concentration of waste, typically higher or equal to 50% (S4, S5, S7, and S9); the thermal conductivity was approximately reduced by almost 25%, while the compressive strength was reduced by 45–60%. One can consider that this zone is rather characterized by a constant thermal conductivity.
- The second zone gathers a moderate concentration of waste (S2, S3, S6, and S8), showing that the compressive strength is rather constant (a reduction of around 25%) whereas the thermal conductivity ratio is reduced by between approximately 5 and 20%. This zone will be considered as a zone with a constant compressive strength.
- Finally, the third zone, corresponding to the low concentration of waste, behaves mechanically as the standard concrete, the only noticeable difference being the lower thermal conductivity.

These results fall into accordance with the results found in the literature and infer the fact that, depending on the type of application, the appropriate concentration of waste aggregate can provide significant advantages in comparison to regular concrete. For example, through adding a low concentration of waste materials, the mechanical properties of the newly formed concrete are similar to those of the regular concrete, being good for the structural elements of buildings or, through adding an increased quantity of waste materials, the mixture provides a lower material conductivity, helping insulate the building and preventing heat loss [22,23,24,25,27,28,29].

## 6. Conclusions

The experimental study investigated the use of waste materials as a partial substitution of aggregates and cement in manufacturing concrete, and analyzed the mechanical properties via the compressive strength, density variations, and the thermal properties via the thermal conductivity parameter.

Three zones of behaviors were highlighted, the first one, corresponding to high waste concentration, leads approximately to a constant reduction in the thermal conductivity. The second, characterized by moderate waste concentration, behaves as a constant compressive strength. In addition, finally, the third one, defined by very low waste concentration, induces only a very small reduction of the thermal conductivity with the compressive strength approximately the same as standard concrete.

By analyzing the compressive strength of the concrete with waste aggregates substitution, the study highlighted that the concrete can be used as a structural material such as self-sustained walls for low waste concentration, the differences in mechanical properties being negligible. On the other hand, high concentrations of waste aggregate in the concrete lead to a decrease in thermal conductivity, providing good solution for nonstructural walls with an increased insulation capacity in comparison to regular concrete walls, preventing heat loss.

One of the most important key factors is that, through reusing the waste and not using conventional aggregates, the carbon footprint of the building is reduced, this being a significant factor in the development of the construction sector in the future.

The studies revealed that a 10% replacement of the cement with fly ash and a 10% replacement of the sand with chopped PET had minimal impact on the properties of concrete, but on an environmental scale, if concrete were to have a portion of the natural aggregates replaced, this would have a significant impact both in terms of the reduction in necessary materials and in terms of pollution, as the waste materials would be given a new purpose.

## Figures and Tables

**Table 1:** Summary of research papers on concrete with waste materials.

NR.	Contents of the Paper	Year	Ref.
1	The study analyzed the substitution of raw materials with fine—fMRA (25% and 50%) and coarse—cMRA (0%, 25% and 50%) mixed recycled aggregates. The study determined that the optimal percentage of an fMRA replacement is 25%.	2021	[22]
2	The paper highlighted a correlation and regression analysis of lightweight concretes with presoakedred ceramic waste aggregate, expanded clay coarse aggregate as waste aggregate and varying degrees of copper coated steel fibre used as reinforcement.	2022	[23]
3	This study analyzed the mechanical properties and durability of medium quality concrete with a replacement of the natural coarse aggregate (NCA) with various percentages of recycled coarse aggregate, RCA, (25%, 50%, 75% and 100%). The results reveal few differences between the concrete with RCA and the reference concrete in terms of the mechanical properties and durability related measurements.	2021	[24]
4	This research involved the experimental investigation of using fly ash admixture collected by a wet process as a replacement of the fine part of the aggregates in concrete. The experimental results show a favourable behaviour of the concretes based on fly ash to sulphate aggressive actions in the first part of life.	2020	[25]
5	The purpose of this paper was analyzing the performance of concrete with granules of corn cob and sunflower with a sodium silicate solution treatement (0%, 20% and 40%). The experimental analysis highlighted that the concrete with vegetal raw materials had a reduced density but, at the same time, a reduced compressive strength as vegetal aggregates have higher water absorption capacity. Sunflower concrete presents superior mechanical strengths in comparison to corn cob concrete.	2020	[26]
6	This study, through a statistical experimental analysis, evaluates the performance of concrete reinforced with recycled PET fibres with various fibre doses and aspect ratios. The results highlighted that the introduction of recycled PET fibres into concrete provides residual strength capacity to the concrete, with a reduced effect on the volumetric weight, ultimate flexural and compressive strength.	2021	[27]
7	The experimental research involved analysing the properties of concrete with sawdust as a replacement of sand containing 0%, 5%, 10% and 15% of sawdust. Through adding sawdust the results showed that the biological oxygen demand increased as the replacement percentage increased, but the compressive strength decreased.	2019	[28]
8	The study researched the properties of fly ash as a basic raw material used in the production of concrete. The inclusion of fly ash in concrete, referenced through multiple studies, provide increased mechanical and microstructure properties in comparison to the use of cement alone.	2020	[29]
9	The paper analyzed the incorporation of waste materials, such as sugarcane bagasse ash (SCBA), metakaolin (MK), and millet husk ash (MHA), and determines their effect on the fresh, hardened properties and embodied carbon of concrete. The study revealed that the use of SCBA, MK, and MHA up to 10–15% separate and combined as ternary cementitious material (TCM) in concrete provides ideal results for structural applications.	2022	[30]
10	The researchers reviewed the impact natural seawater has on the properties of concrete. The study points out that, in the long term, seawater is harmful to the concrete structures built in marine environments and that, through the use of supplementary cementitious materials (SCMs) such as rice husk ash, coal bottom ash and blast furnace slag in the concrete mix, the degradation is reduced. Another interesting fact is that, through adding seawater during the curing and mixing of concrete, the tensile properties of the concrete are increased.	2021	[31]

**Table 2:** Concrete mixture with waste substitution.

	Materials								
Concrete Sample		Sand		Rocks (4–8 mm)		Rocks (8–16 mm)		Cement	
		Percent	Volumic Mass	Percent	Volumic Mass	Percent	Volumic Mass	Percent	Volumic Mass
S0	natural aggregate	100%	803.16	100%	384.12	100%	558.72	100%	360
	waste substitution	0% PET		0% PET		0% PET		0% fly ash	
S1	natural aggregate	90%	722.84	100%	384.12	100%	558.72	90%	324
	waste substitution	10% PET		0% PET		0% PET		10% fly ash	
S2	natural aggregate	80%	642.52	100%	384.12	100%	558.72	90%	324
	waste substitution	20% PET		0% PET		0% PET		10% fly ash	
S3	natural aggregate	60%	481.9	100%	384.12	100%	558.72	90%	324
	waste substitution	40% PET		0% PET		0% PET		10% fly ash	
S4	natural aggregate	40%	321.26	100%	384.12	100%	558.72	90%	324
	waste substitution	60% PET		0% PET		0% PET		10% fly ash	
S5	natural aggregate	100%	803.16	40%	153.64	100%	558.72	90%	324
	waste substitution	0% polystyrene		60% polystyrene 4–8 mm		0% polystyrene 8–16 mm		10% fly ash	
S6	natural aggregate	90%	722.84	80%	307.29	100%	558.72	90%	324
	waste substitution	10% polystyrene 0–4 mm		20% polystyrene 4–8 mm		0% polystyrene 8–16 mm		10% fly ash	
S7	natural aggregate	80%	642.52	50%	192.06	100%	558.72	80%	288
	waste substitution	20% polystyrene 0–4 mm		50% polystyrene 4–8 mm		0% polystyrene 8–16 mm		20% fly ash	
S8	natural aggregate	90%	722.84	100%	384.12	100%	558.72	90%	324
	waste substitution	10% saw dust		0% saw dust		0% saw dust		10% fly ash	
S9	natural aggregate	60%	481.9	100%	384.12	100%	558.72	90%	324
	waste substitution	40% saw dust		0% saw dust		0% saw dust		10% fly ash	

## Data Availability

All data are contained within the article.

## Nomenclature

PET	Polyethylene terephthalate
V	The volume of the specimen [m^3^]
m_a_	The mass of the specimen in air [kg]
m_st_	The apparent mass of the immersed stirrup [kg]
m_w_	The apparent mass of the immersed specimen [kg]
ρ_w_	The density of water, at 20 °C [kg/m^3^]
ρ_c_	The density of the specimen [kg/m^3^]
m	The mass of the specimen [kg]
t_d_	The diffusive time of the sample
e	The sample thickness [m]
a	Thermal diffusivity [m^2^/s]
b	Thermal effusivity [Jm^−2^K^−1^s^−0.5^]
k	Thermal conductivity [W/mK]
C_p_	Thermal capacity [J/kgK]
x	The ratio of the sample
